# The body social: an enactive approach to the self

**DOI:** 10.3389/fpsyg.2014.00986

**Published:** 2014-09-12

**Authors:** Miriam Kyselo

**Affiliations:** Department of Logic and Philosophy of Science, University of the Basque CountryDonostia-San Sebastián, Spain

**Keywords:** enactive self, social self, embodied self, body-social problem, distinction and participation

## Abstract

This paper takes a new look at an old question: what is the human self? It offers a proposal for theorizing the self from an enactive perspective as an autonomous system that is constituted through interpersonal relations. It addresses a prevalent issue in the philosophy of cognitive science: the body-social problem. Embodied and social approaches to cognitive identity are in mutual tension. On the one hand, embodied cognitive science risks a new form of methodological individualism, implying a dichotomy not between the outside world of objects and the brain-bound individual but rather between body-bound individuals and the outside social world. On the other hand, approaches that emphasize the constitutive relevance of social interaction processes for cognitive identity run the risk of losing the individual in the interaction dynamics and of downplaying the role of embodiment. This paper adopts a middle way and outlines an enactive approach to individuation that is neither individualistic nor disembodied but integrates both approaches. Elaborating on Jonas’ notion of needful freedom it outlines an enactive proposal to understanding the self as co-generated in interactions and relations with others. I argue that the human self is a social existence that is organized in terms of a back and forth between social distinction and participation processes. On this view, the body, rather than being identical with the social self, becomes its mediator.

## INTRODUCTION

Models and conceptions of the self are diverse. It is considered a substance or a thing, a concept, a narrative, a system, a process or a function; some even argue that there is no such thing as the self (Hume, 1739; James, 1890; Dennett, 1992; Hayward, 1998; Tani, 1998; Perlis, 1999; Strawson, 1999; Dainton, 2004; Metzinger, 2004; Zahavi, 2008). This list is not exhaustive but it makes a point: there is no unifying concept of *the* self.

The lack of a coherent concept of self is not merely a philosophical armchair problem but remains an issue of general theoretical, as well as practical, concern. Here lies the main motivation for the present paper: to propose avenues for a philosophy of self that eventually aids in facilitating dialog and research on the self across the disciplines in cognitive science.

One desideratum for a cross-disciplinary approach to the self is that it acknowledges the diversity of phenomena associated with self and does not make an essentialist claim according to which the self is, for example, either neurological or phenomenal while other aspects are seen as irrelevant or added on. Shaun Gallagher has recently warned against such reductionism of understanding *the* self as essentially this or that “and nothing more.” Alternatively, Gallagher proposes a pluralistic, so-called “pattern theory of self:”

[W]hat we call a “self” is a cluster concept which includes a sufficient number of characteristic features. Taken together, a certain pattern of characteristic features constitute an individual self. (…) I propose that we think of these aspects as organized in certain patterns, and that a particular variation of such a pattern constitutes what we call a self. (Gallagher, 2013, p. 2)

Examples of aspects that could serve as constituents of a self-constitutive pattern are *minimal embodied*, *minimal experiential*, *affective*, *intersubjective*, *psychological/cognitive*, *narrative, extended,* and *situated*. According to Gallagher, adopting a pattern view of self helps understanding different aspects of the self non-reductively “as compatible or commensurable instead of thinking them in opposition.” He illustrates this for a particular conceptual tension in cognitive science, namely the question whether self-hood is best explained in terms of cortical midline structures, a particular brain region (Northoff and Bermpohl, 2004) or whether the necessary condition of self-hood is not rather that all experiences acquire a first-person perspective (Légrand and Ruby, 2009). On Gallagher’s pattern approach, resolving this conceptual tension is now pretty simple: do not reside with either of the positions but allow for the 1st person perspective or particular neuronal activation patterns to each count as one “among other aspects” (Gallagher, 2013) of an organized pattern of self – which in the present case, is a pattern defined in terms of minimal embodied and experiential aspects.

I agree with Gallagher’s pledge for pluralism, but I also think that his radical openness might prove somewhat too laissez-faire: what makes any of the listed features part of a (meta-)theory of self and what is it that makes a pattern of self acquire its particular organization? Once the diversity of self related phenomena is acknowledged, we also need to understand how the elements of a collection of relevant self features interrelate.

A pattern approach to the self acknowledges diversity but lacks integration, offering no account of the individual as explanatory whole. This poses more than a philosophical armchair problem because what researchers in cognitive science believe the self to be impacts very practically the way they conduct research, from the choice of methodology in setting up experiments and forming hypothesizes, to the interpretation of results. It affects how a medical doctor assesses a person’s state of consciousness and well-being or how a psychologist conceives of pathologies of the self and thus whether she choses to treat with pharmaceuticals, body therapy or social and dialogical intervention.

Understanding the self should therefore not consist only in composing lists of aspects according to standards of a given contextual convenience; we still need a notion of the self as a whole, something that can count as a distinguishable unit of explanation and eventually help to interrelate different aspects of the self. As Olson had argued almost two decades ago:

Simply extending the list will only make matters worse. What we need is not just an account of self that would command wider assent than any of these, but one that would synthesize them and show them all to reflect a part of some larger, common idea (Olson, 1998, p. 651).

What I suggest in this paper is that such a larger, common idea exists and that we do not have to chose between either a pluralistic and laissez-faire or an essentialist and reductive approach to the self. A middle way, that acknowledges diversity, while also offering an integrative perspective on the self as a whole could be found in considering the self from the perspective of enactive cognitive science.

The enactive approach holds that biological and mental phenomena are continuous, which means that it characterizes the identity of cognitive beings by similar principles and concepts as the identity of living beings (Clark, 2001; Thompson and Varela, 2001; Di Paolo et al., 2010). It proposes the biologically based concept of autonomy to capture cognitive identity in terms of self-generated, self-determined precarious networks (Thompson, 2007; Di Paolo et al., 2010). The concept of autonomy has a fruitful link to the question of self since both are at heart about *individuation* and concerned with understanding what makes something – or, in the present case, somebody – a coherent unity. The enactive perspective on identity is neither reductionist nor essentialist but aims at a wide enough focus to accommodate the diverse aspects of cognition, while still being concise enough that it can provide constraints to interrelate them. For that reason I utilize the concept of autonomy to inspire a new perspective on theories of self. In this enactive approach, I take the fact that human life is genuinely social to be of crucial relevance. I argue that humans live not only in a world of others that affect them and that they relate to, but that *qua* being interactors in a social world, they also co-constitute each other’s self. The human self is not only saturated by the social, but is also entirely inconceivable without it.

The paper involves two layers of novelty, first, it provides an elaboration of the notion of autonomy and the higher levels of the life-mind continuity axis, which moves from basic, sensorimotor cognition to psychological and socially mediated forms of human (cognitive) individuation. Second, it promises to help clarify current conceptual tension associated with the bodily and social dimension of self: while embodied cognitive science has recognized for a while that humans are not their brains but rather embodied and situated social beings, the field still faces another dichotomy, namely the split between individual selves and the social world of others. The social still plays the role of an outside and divided context: the external, independently given world into which these newly embodied, yet essentially isolated selves parachute^1^.

The following elaborations of the enactive concept of autonomy are thus at the same time concerned with what I call (in reminiscence of the body-mind problem or as a successor to the body-body problem) the body*-social* problem, i.e., the question for philosophy of cognitive science about how bodily and social aspects figure in the individuation of the human individual self as a whole (Kyselo and Di Paolo, 2013)^2^.

The strategy for this paper is as follows: I begin by laying out the body-social problem. This is followed by an introduction to the enactive approach to cognition, focusing particularly on the notion of autonomy. In the next section I show that a version of the body-social problem also applies to recent work in enactive approaches to social cognition, in particular to participatory sense-making. Coming back to the logic of some early enactive philosophy by Hans Jonas, I then elaborate the notion of autonomy in terms of sociality and outline an enactive approach to the self that acknowledges diversity without being essentialist and reductive. Support for this proposal is provided considering empirical evidence from research on social pain, quality of life reports in global paralysis, as well as some examples from everyday life.

## THE BODY-SOCIAL PROBLEM IN COGNITIVE SCIENCE

There is a conceptual problem arising for recent philosophy of cognitive science. It has to do with two important advances in the development of cognitive science and how they relate to the human self- firstly, the realization that cognition is not brain-bound, but embodied (the “embodied turn”) and secondly, the increasing awareness that cognition is not individualistic, but also social (the “social,” or if you will, “interactive turn,” De Jaegher et al., 2010). Each of these developments itself constitutes an answer to a previously noted conceptual dichotomy: the embodied turn concerned the dichotomy between brain and body, and the social turn, the gap between individual and others.

Let me explicate this tension beginning with the first insight that cognition is not in the head. Recent embodied and situated cognitive science seeks to overcome the brain-bound view of cognition and thereby the clear-cut separation between the individual cognitive system and the environment as an objective and independent given. Cognition is now considered a dynamic interplay of individual bodily and environmental processes, with the brain as a mediator of that interplay (Fuchs, 2011). In this view, cognition also entails subjectivity so that research on cognition is no longer restricted to third-person operational descriptions but also relies on subjective and phenomenological observations from a 1st and 2nd person perspective (Varela et al., 1993; Lutz, 2002; Lutz and Thompson, 2003; Petitmengin, 2006).

The embodied view in cognitive science has implications for understanding the self. While there are still some people who argue that self is found in the brain (e.g., Feinberg and Keenan, 2005; Churchland, 2013), there now is a much wider range of research on the embodied self that explores the role of more than neuronal bodily structures and action for human identity (Gallagher, 2000; Fuchs et al., 2010). It is investigated as a subjective and experiential bodily self (Zahavi, 2008). There are new investigations on the foundations of self and self consciousness in terms of bodily processes, i.e., sensorimotor structures (Légrand, 2006; Gallese, 2014). The idea that the self is embodied has thus found increasing acceptance.

As a consequence, we see new proposals for understanding disorders of the self (such as autism, schizophrenia, etc.) not simply as neurological dysfunctions, but rather as disturbances of sensorimotor capacities of this bodily subjectivity. Accordingly, there are also suggestions for new forms of body based treatment and therapy (Fuchs, 2005; Drayson, 2009; Röhricht, 2009; Parnass and Sass, 2010). Perhaps here it is most evident why cognitive scientists cannot merely adopt a pattern approach to the self, as Gallagher suggested. Explaining schizophrenia as a disorder of the embodied self, for example, cannot imply that the ordered self is considered to be a lose collection of neuronal, social and also bodily aspects. The way we reason for example about what goes wrong in a disorder of the self reveals that instead we already have implicit assumptions about what counts as the ordered self *as a whole*, a coherent explanatory unit – the body, in the present case.

While these considerations are not exhaustive, it thus seems fair to say that cognitive science is on a good track to move from the brain-bound to the embodied view of the self, where embodiment amounts to more than a conceptual add-on.

Consider now the second development in cognitive science: the growing acknowledgment of the idea that cognition involves the social and is, broadly construed, also concerned with intersubjectivity and with understanding others. This has become a subject of interest across the disciplines in cognitive science. The relevance of social interaction is, for instance, argued for in psychological studies on child development, particularly in neo-natal imitation and early infant–mother relations (see e.g., Trevarthen and Aitken, 2001; Reddy, 2003; Rochat et al., 2009). The interpersonal approach has attracted increasing interest in neuroscience, in particular with regards to the question of understanding others, e.g., in research on the (in)famous mirror neurons (Gallese and Goldman, 1998; Gallese, 2013), and in simulation theory approaches (Frith and Frith, 2010; Gallotti and Frith, 2013). In more philosophical approaches we find the corresponding objections to brain-based accounts of social cognition (e.g., Gallagher, 2001) and developments emphasizing the social dimension of self in terms of narrative practices (Hutto, 2010, 2014). There have also been more general considerations about the relation between low-level embodied and social forms of cognition (De Jaegher and Froese, 2009) and new basic concepts that capture the essential role of intersubjectivity in structuring human cognition (De Jaegher and Di Paolo, 2007). In addition, we observe a flowering dialog between cognitive science and phenomenology of intersubjectivity reconsidering authors such as Husserl, Merleau-Ponty, Gurwitsch, or Schütz (e.g., Thompson et al., 2005; Zahavi, 2008).

The question is, how do these two developments, the embodied and social, go together; or better, how do the bodily and social dimensions figure in the individuation of the human self? From a pattern theory approach to the self à la Gallagher they seem compatible and could complete existing theories of the self, adding novel (e.g., sensorimotor and sociocultural) items to a list of (previously neuronal) aspects associated with the self. This perspective is mainly descriptive, which is why it also risks not adding much to understanding the self from a philosophical point of view. As already pointed out in the introduction, one of the reasons why it matters that we do adopt more than a mere completion perspective is that (interdisciplinary) research cannot do with a lose collection of aspects, but must refer to a coherent unity, with which particular aspects, such as neuronal, bodily or social are then possibly associated.

I therefore suggest considering that embodied and social approaches to cognition entail the attempt to re-determine the boundaries of the individual. From this perspective, the embodied and social turns would therefore entail claims about what counts as the individual (agent, system, person, self) as a whole, each specifying an *individuating principle* or the essential or minimal sense of this whole.

However, upon accepting that embodied and social cognitive science makes implicit assumptions about what counts as the individual in this sense, we will see that these developments are, as it were, in tension. The self as a whole can either be embodied or social, but it cannot be both.

Cognitive scientists might give one of the following two answers in response to this. According to the first, they might assume that the body is equated with the self. When speaking of the individual, then clearly no longer referring to the brain, they mean the lived and living *body* as a whole. According to this, there is an embodied core self, which is equated with the individual embodied or living organism (Parnass and Sass, 2010, p. 230). Other recent approaches associated with the idea of such an embodied core self are, for example, Albahari’s (2007) concept of *perspectival ownership*, Damasio’s (2006) *core consciousness* and Zahavi’s (2008) *minimal self*, which considers self from a phenomenological viewpoint of bodily subjectivity. It is assumed that such a bodily, minimal self is present from birth (Krueger, 2011).

Even though proponents of this answer (the self is equal to the body) would probably agree that embodied and social aspects of self are closely interrelated, there seems to be a strong intuition that something about the self remains entirely independent from the question of sociality (Zahavi, 2008, 2010) and that this something – a core self, if you will – can be associated with the body as an organic, separate and individual entity. The social in this version is of course not irrelevant, yet because it provides the context in which the minimal bodily self is embedded, it figures non-constitutively in the individuation of self^3^. In other words, there can be a self as a whole without the social. I call this claim about the interrelation of body, social and self the *social as contextual* claim.

The other way to answer the question of how social and bodily dimensions relate with regards to the individuation of self as distinguishable unity is to assume that the social, instead of the body, is the primary source of individuation. One might call this the *social as constitutive* claim. It states that the core self relies on social processes and that it could not be a self without them. On this account, in its most minimal sense, the self is not neuronal or bodily, but must be essentially a social self.

There are not many researchers in cognitive science who would currently adopt this position decisively, a notable exception being De Haan (2010) who criticized the notion of a *minimal bodily* self and claimed quite specifically that the self, in its minimal sense, is a social self. The idea of self as social is of course not new; it can be traced back to the work of researchers such as Mead (1934), Buber (1947/2002), Vygotsky (1986). Hermans et al. (1992) suggested over a decade ago that the self is social and dialogical in the sense that “other people occupy positions in the multivoiced self”. However, it is not clear whether these approaches make an essentialist/constitutive or a contextual claim about the role of the social for the self. In order to argue for a *constitutive* role of the social in the individuation of the self as a whole no stronger statement about the status of the body might be required as it leaves the possibility open that the relevant processes of self individuation could be mediated in terms of mere brain activity, thus trivializing the role sensorimotor structures and other non-neuronal bodily structures. Hermans and Gieser (2012), for instance, locate the biological basis of the dialogical self in the orbitofrontal cortex and the subcortical limbic system, thus leaving the relation between self as bodily and self as social underspecified (Cresswell and Baerveldt, 2011). An emphasis on the role of the social in the constitution of the self as a whole might therefore risk to downplay the other achievement in cognitive science, the embodied turn.

It is possible to make a stronger statement about the role of the body for an essentially social self. But for a claim that the body plays a non-trivial role in the social constitution of the self as a whole to make sense a clarification is required on what counts as a body. That is because embodiment, commonly understood, still equates with organismic embodiment as well as with movement (for a discussion see Kyselo and Di Paolo, 2013), and there is nothing social about the organismic or the moving body *per se*. Nevertheless, whether or not the essentially social self is seen as neurally or bodily mediated, it would still be in tension with the contextual social contribution claim according to which the body is the primary source of individuation.

This is the prevalent tension in cognitive science with regards to the individuation of self. In reminiscence of the body-mind problem or as a successor to the body-body problem I will call it the *body-social problem*, i.e., the question for philosophy of cognitive science about how bodily and social aspects figure in the individuation of the human individual self (Kyselo and Di Paolo, 2013). This tension exists for any approach in cognitive science making a claim about the self as a whole or coherent unity, thus implying a more-than-pluralistic notion of the self. Proponents of an embodied view of individuation risk giving lip service to the social while those emphasizing the role of the social risk doing the same with respect to the body. Both approaches are mutually exclusive. Without due conceptual clarification, adopting either version, i.e., a primacy of embodiment or a primacy of the social, reduces the other. The assumption that the body individuates the self while the social remains merely context puts into doubt the second disciplinary development in cognitive science, the social turn, and would reinvite accusations of methodological individualism. One could argue that while now there no longer exists a dichotomy between the brain as individual and the world of others, there still exist a dichotomy between the body-as-individual and the world of others. Yet it remains unclear how to work an embodied perspective into an account that takes seriously the role of the social in individuation, when the relevant contribution could equally be made by the brain.

To see that this body-social problem is not an abstract theoretical issue, consider two empirical examples: social pain and locked-in syndrome. Firstly, Eisenberger (2011) has shown that the experience of social rejection (in her example, being excluded from participating in a game) leads to the same activation of neuronal circuitry as physical pain (in reaction to increased temperature). This arguably suggests that people who are socially rejected experience this as similar distressing as bodily pain. Eisenberger argues that this has evolutionary reasons. Humans rely on “social connection” in order to ensure their survival. Social rejection hurts so we avoid (life) threatening situations in which we find ourselves separated from others. Here it seems that the body constitutes the core of human existence as a biological whole. Through pain signals it ensures its integrity, while the social is a means to the same end.

Secondly, consider locked-in syndrome, a case of global paralysis, which leaves a person’s entire body paralyzed (with the exception of minimal eye movement, such as blinking), yet her consciousness preserved. The patient’s bodily capacities are drastically restricted. Yet inquiries about the quality of life in patients with locked-in syndrome reveal that their self-reported well-being does not differ significantly from that of “normal” subjects. These studies show that the patients’ well-being is not equated with physiological capacities. What mattered is that that they were able to engage with others, be recognized and experience themselves as subjects. Locked-in syndrome was not considered a physiological but rather social condition (Gosseries et al., 2009; Lulé et al., 2009). These findings seem counter-intuitive for an embodied approach to the self. If the self was equated with the body and the bodily self is what grounds first-person subjectivity then the patients’ well-being should be worse, since locked-in syndrome affects the body as a whole.

How we interpret these empirical examples will in each case depend on which version of body-social relation to the self we adopt. Should we explain bodily experiences (such as social pain) and self experience (positive quality of life in LIS) using a theory of the self seated in bodily or organic processes or do these cases rather show that human nature and thus the self is genuinely social and that the body plays an important, but rather enabling role?

One option to avoid a pluralistic or pattern approach to the self (in which body and social co-exist as different aspects of the self) and to still provide an alternative for a cross-disciplinary approach to the self, is to adopt an essentialist perspective, according to which the self as a whole is either embodied or social. But this option risks privileging one dimension, while reducing the other to a contextual element. Either view remains problematic for the purposes of cognitive science. A pattern approach acknowledges diversity without integrating, while an essentialist view offers a sense of unity but at the risk of being reductive and of trivializing the role of either the social or embodied turn in cognitive science. Does this mean we have to decide that one of the two is less relevant or are merely dimensions of a lose pattern of self?

I do not think so. There is a way to argue for a more than pluralistic perspective that does not require one to assume an essentialist perspective on the self as being *either* embodied *or* social. I propose that the body-social problem can be resolved by adopting an enactive approach to the self. However, this point requires nuance and elaboration, since I think there is a version of enactivism that does address the role of bodily and social processes in the emergence of individual autonomy – namely, participatory sense-making – yet still gets us into the same trouble with the body-social problem.

## THE ENACTIVE APPROACH TO COGNITION

A central proposal of the enactive approach is that there is a continuity of mind and life, i.e., that mental phenomena can be understood based on the principles that describe the organization and behavior of all life, including the simplest life form such as the single cell organism (Varela, 1997; Thompson, 2007). The philosopher of biology Hans Jonas provided some of the basic definitions of living and cognitive identity. They have been taken up by more recent research in the enactive tradition. The most important idea with respect to the present paper concerns how Jonas conceived of the relation between the individual organism and the world. According to Jonas, the boundary, i.e., that which allows us to identify the individual organism *as* individual is an emerging distinction. He says:

Sameness, while it lasts … is perpetual self-renewal through process, borne on the shift of otherness. This active self-integration of life alone gives substance to the term “individual” … its very existence at any moment, its duration and its identity in duration is, then essentially its own function, its own concern, its own continuous achievement (Jonas, 1966/2001, p. 80).

Crucial to Jonas’ idea is that the processes involved in the emergence of the organism are in principle not different than those of the organism’s environment. These organic processes have a “double nature:”

the materials are essential to [the organism] specifically, accidental individually; it [the organism] coincides with their actual collection at the instant, but is not bound to any one collection in the succession of instants ... “[d]ependent on their availability as material, it [the organism] is independent of their sameness as these; its own, functional identity, passingly incorporating theirs, is of a different order (ibid.).

This means that the individual organism creates its identity as an organism by negotiating a permanent tension between a need for material resources from the world that “it is made of” and the simultaneous drive to emancipate or free itself from *some* of the material processes, so it can exist as an independent individual. The organism’s identity thus relies on organic matter that serves as “constructive material” on one side, and yet at the same time provides identity by being organized in a particular functional way (“a different order”). A fundamental tension exists at the heart of organic life, between a general dependence on material resources and a striving for emancipation from them. Jonas called this tension “needful freedom” (Jonas, 1966/2001, p. 80).

Needful freedom means that an organism’s identity is ontologically relational and interactively constructed. Jonas sees the organism as a precarious being, remaining restless as long as it is alive. As Thompson has put it, the “organism has to change; stasis is impossible” (Thompson, 2007, p. 152). It is concerned with its own survival and with having to avoid conditions that lead to disintegration, i.e., its death. The organism is thus permanently in need because in order to survive it has to continuously interact with the environment. One can say that the organism is therefore relatively, but never fully, “in control” of the construction of its very identity.

Over the last decades Jonas’ ideas have been elaborated and more formally expressed in various ways, which together ground an enactive view of cognitive individuation (e.g., Maturana and Varela, 1980; Varela et al., 1993; Varela, 1997; Weber and Varela, 2002; Di Paolo, 2005; Thompson, 2007; Di Paolo and Thompson, 2014). The basis for this view is the notion of *autopoiesis*, according to which living beings are defined as self-organized autonomous networks that produce and sustain themselves as a systemic whole – an *identity* within a particular domain (Varela, 1997; Maturana and Varela, 1980, 1987). The production and maintenance of such an identity requires that some relations between the processes of the network remain constant despite structural dependence on the environment. This characteristic of identity has been referred to as *operational closure* (Maturana and Varela, 1987; Di Paolo et al., 2010). More recently these ideas have been elaborated in order to understand not only biological but also cognitive individuation. Some enactivists propose that cognitive systems are best conceived as *autonomous* systems. According to this idea, a cognitive system’s identity is a network of processes that self-produces and maintains the network as an *inter*connected network, i.e., each process in the network is not only enabling but also enabled by some other process. The identity is sustained under “precarious conditions,” since without being organized in an interconnected way the individual processes making up the network would risk running down and the network as a whole could dissipate (Di Paolo and Iizuka, 2008). In line with Jonas, from the enactive perspective cognitive beings are thus considered intrinsically purposeful beings: they strive to maintain life, which is considered a natural property (Weber and Varela, 2002).

Based on this concern for survival, cognitive beings develop a perspective on the world, from which environmental features and interactions are evaluated and acquire a meaning and a normative status. Not every aspect of the world matters. The normative status of environmental aspects and interactions depends on whether they count as threatening or beneficial to the basic goal of identity maintenance (Di Paolo, 2005; Thompson and Stapleton, 2009). Here lies, according to more recent proponents of the enactive approach, the difference between a mere living system and a living cognitive system. A cognitive system’s perspective on the world depends not only directly on its physical survival – the “mother-value of all values” (Weber and Varela, 2002, p. 111) – but enlarges its action possibilities, from immediate reactions to existential perils, to a recognition of more fine-grained ways to maintain its existence. A cognitive system evaluates its interactions *adaptively*, thus flexibly regulating and changing its own conditions of identity maintenance (Di Paolo, 2005). Cognitive individuation in the autonomous self-production of identity entails a view of cognition as goal-directed, value-driven and purposeful. Cognitive systems have a basic intrinsic twofold goal: to create and maintain an identity and to generate sense or meaning.

For that reason cognitive identity of the autonomous system cannot only be grasped from a third-person, operational definition of the processes involved in its individuation; instead, it requires a view from which the world is encountered and interactions are evaluated *by the system* itself. The enactive approach thus adopts a complementary perspective on cognition, one which also considers the perspective of the cognitive system itself. On this view, research on cognition also relies on subjective and phenomenological observations from 1st and 2nd person perspectives (Varela et al., 1993; Lutz, 2002; Lutz and Thompson, 2003; Petitmengin, 2006). With regard to the question of the self, this means taking the first-person perspective – and therefore subjective experiences and phenomenological investigation – seriously, when it comes to describing its basic structures.

## THE BODY-SOCIAL PROBLEM IN ENACTIVISM

Let me now consider how the aforementioned two shifts in contemporary cognitive science, the embodied and the social turn, are accounted for in current work in the enactive tradition. In the enactive approach, the body is what grounds a cognitive system’s identity and individuates it as a living entity. It allows the autonomous system to differentiate itself from the environment (Di Paolo and Thompson, 2014) and it is also the means and reason for the cognitive system’s engagement and evaluations of its interactions with the world (Kyselo and Di Paolo, 2013). On the one hand, the body is assumed to inform the cognitive system how it is faring with regards to its own intrinsic goals – for instance through emotions (Colombetti and Torrance, 2009; Colombetti, 2014) – but it is due to being embodied that, on the other hand, a cognitive system can have any goals at all. If bodily existence were not finite, nothing would matter to a cognitive system. The individuation of identity and sense-making – the adaptive regulation of interaction with the world – can be realized in various ways, including, for example, through the appropriation of non-physiological tools (Kyselo and Di Paolo, 2013). This is based on the life-mind continuity hypothesis, according to which autonomous self-individuation is not limited to biological processes but can be found at higher levels of cognition, too. This brings us to the second shift in contemporary cognitive science, the question of how, for researchers in the enactive tradition, the social figures in the individuation of cognitive identity.

That human life is not merely biological but also social has been taken seriously by some proponents of the enactive approach (Gallagher, 2001; Thompson, 2007; De Jaegher and Froese, 2009; De Jaegher et al., 2010; Di Paolo et al., 2010). One central example for this that I focus on now is “participatory sense-making” (De Jaegher and Di Paolo, 2007). Participatory sense-making reflects a classical idea from sociology and system theory, that based on the dynamical behaviors of (at least two) individual agents an interaction process emerges that exhibits new properties irreducible to the individuals concerned, so that it can be described as a new systemic entity (Luhmann, 1992). It uses the concept of autonomy to characterize this new systemic entity as a *social* form of autonomy, an “interactive autonomy.” Participatory sense-making elaborates on the idea that identity is not passively given but brought forth through interactions with the environment. But it is concerned with a form of autonomy in the relational processes based on coordinated *social* interactions of participants (De Jaegher and Di Paolo, 2007; Colombetti and Torrance, 2009, p. 32).

Recent proponents in the enactive tradition acknowledge that human cognition is not brain-bound, but a matter of embodied, sensorimotor engagement with the environment, as well as a matter of social interactions, as the example of participatory sense-making shows.

But with regards to the present issue, the body-social problem, I show now that the enactive approach currently entails an ambiguity about the role of social interactions for the individuation of identity. To explain this requires, as a first step, to disentangle two senses, in which social interactions appear to be relevant for proponents of participatory sense-making. Firstly, in that social interactions matter with regards to a group of (classically two) individuals, jointly creating the autonomy of the interaction process. Here we look at an autonomous system whose identity as a whole is defined in terms of human social interactions. It is a group identity. Secondly, participatory sense-making also says something about the role of social interactions for the individual: they enlarge individual cognitive capacities.

Participatory sense-making thus implies that there are individuals involved in social interaction. But what can be said about their nature as individual identities? This question remains implicit within the theory. But let me point out some indications of what could count as an answer to what the individual is for participatory sense-making. There are at least two possible readings. One option would be to say that social interactions matter not only for augmenting the individual’s cognitive capacities but also for its identity as such. This seems to be what De Jaegher and Di Paolo (2007, p. 492) have in mind when they write that their “perspective bypasses the circularity that arises from pre-conceiving individuals as ready-made interactors. Individuals co-emerge as interactors with the interaction.” In this vein one might characterize the individual identity as essentially a (socially) relational and interacting being, in other words, as a participant.

This however, raises a worry. Critics might argue that an identity that is defined as being relational or a participant in social interactions runs the risk of dissolving in the interaction process, effectively becoming invisible *as* an individual identity (Hutto, 2010). But why should a focus on the interaction dynamics imply that there is no individual contribution or that the individual risks dissolving? One reason could be that as of now in participatory sense-making the individual’s nature as a relational being is underdetermined with respect to *its own identity*. It appears that the intrinsic purpose of participatory sense-making is not directed at the emergence and maintenance of the individual’s identity but at that of an overall interaction dynamics, in other words, at the group identity. From this perspective, the individual participants of course make an important contribution. They act as constituents of a higher order dynamics, in that they “sustain the encounter, and the encounter itself influences the agents and invests them with the role of interactors” (De Jaegher and Di Paolo, 2007, p. 492). The problem is that if being relational means being part of a process whose properties are irreducible to the individual (p. 494) and if, as De Jaegher and Di Paolo (2007, p. 493) say “the regulation is aimed at aspects of the coupling itself so that it constitutes an emergent autonomous organization,” then for the individual to be an individual it would have to adapt to an external norm. This norm has to do with the groups identity and the interaction dynamics of which the individual is part. If that were generally be the case, then the individual would actually not be autonomous but rather heteronomous, as it is not governed by its own laws of self-organization. The individual would risk dissolving because it merges with the social environment rather than emerging from it.

Note that De Jaegher and Di Paolo (2007, p. 492) try to avoid the worry that identity is lost in interaction dynamics. They emphasize that “the autonomy of the individuals as interactors must also not be broken … [o]therwise the individual (as the other) would “become a tool, [or] an object.” They appear to defend their view by saying that a person is individuated from others *qua* being *embodied.* This is supported by quotations such as the following: “When we speak about cognitive agents in interaction, the basis for such a coupling can take various shapes and involve various perceptual systems, sensorimotor flows, neural, and physiological processes, external objects, and technological mediation.” Co-regulation involves “bodily variables, such as relative positions and timing between movements, coordination between perceptual systems, and neuro-physiological variables” (ibid.). Such wording suggests that the individuals involved in participatory sense-making are bodily beings. If the mentioned processes and mechanisms of co-regulation ground the individual’s identity then it would be an individual that moves, has a brain, interacts with material environment, in short is a body. However, it would also be, as it were, unsocial because nothing in the definition of the body as such is social. The identity of the individual is then defined not in social terms, but remains bodily. Ironically, in their very attempt to keep the individual from dissolving, participatory sense-making therefore risks to downplay the role of the social. The body, while differentiating the individual from others, would be a locus of isolation, not a means of connection and engagement.

One way for proponents of participatory sense-making to avoid this second horn of the dilemma would be to admit that individuation of human identity is not fully determined in terms of bodies in isolation but requires that the body engages in *socially mediated* interactions with the world. This would permit a view according to which both claims come together: individuals are not merely embodied, but they are also interactors. This may be the view that proponents of participatory sense-making are actually arguing for. However, this position would suffer from the first horn of the dilemma of the body-social problem, for it implies that the social matters only as a context, in which bodily individuals relate to each other as otherwise ready-made identities. Participatory sense-making risks trivializing the role of social interactions as mere context, a position that stands in stark contrast to the original claims of the theory.

When it comes to defining the individual, the enactive approach currently thus gives an ambiguous answer to the body-social problem. With regards to embodiment and the role of social interactions for the self as a whole, it remains caught in a dilemma. With its identity heteronomically defined as being a participant, the individual either risks immersing and getting lost in the social interaction, or the individual becomes isolated, with its identity defined in terms of bodily processes. Like other research in embodied and social cognitive science that attemps to define the individual as a whole, participatory sense-making actually runs the risk of being individualistic, not in the sense that it implies a split between an objective and material world and the brain-bound individual, but rather a split between a material and social world and *body-bound* individuals.

To conclude, while participatory sense-making is essential for understanding social cognition as a processual and interactive phenomenon and will be important to understand some of the underlying dynamics of group identity construction and interrelations of individuals, its concept of the individual remains ambiguous. We have still to provide more steps within the conceptual move from the low-level cellular to the higher, bodily and social levels of autonomy.

Without further conceptual clarifications and a definition of what counts as the individual, the concept of autonomy, which is considered a crucial building block for the enactive approach to human cognitive individuation, remains underspecified. If this remains the case, critics of the enactive approach might find it difficult to see how the notion of autonomy can help cognitive scientists address important questions at the intersection of individual and social cognition.

As we will see in the remainder of this paper, the notion of individual autonomy can be elaborated following the classical logic of the enactive position itself. In the next two sections I outline how one can account for the individual in cognition in a way that avoids the body-social problem without being reductive or essentialist. I propose an approach to the self that acknowledges plurality while also offering an idea how it might form a coherent unity.

## AN ENACTIVE APPROACH TO THE SELF

In this section I outline an account for the individual self in a way that avoids a tension between the role of bodily and social processes in cognitive individuation. From an enactive point of view, it is therefore crucial to carefully distinguish on the one hand between two different kinds of identity that the enactive approach refers to as autonomous system – the identity of a group (autonomy in the interaction process) and the identity of the individual (individual autonomy). On the other hand, we also need to differentiate two kinds of organizational principles – one in terms of bodily and organismic, and the other, in terms of social interaction processes. What I focus on here is how bodily and social processes matter for individual autonomy.

We must also acknowledge that, while the individualism entailed in an (essentially) embodied view of the self is reductive, it also has an important point: it introduces a distinction between the individual and the world and thus makes it distinguishable as what it is: an individual, and not the world. As I argue in the following, it is not the distinction between individual and world *per se* that we should give up, but the degree to which a brain- or body-bound view would force us to endorse it. Speaking about separation from the environment (and thus about the individual as an identifiable whole) does not rule out that social interactions are vital for cognition (as participatory sense-making has it) nor force us to assume that the individual is an isolated being parachuted into the social world. The solution is to reconcile both views by finding a common ground from which a middle way can emerge.

I propose that this common ground can be derived from the logic of the individuation of organismic identity entailed in Hans Jonas’ notion of “needful freedom.” The notion captures a principle that I believe is most useful for beginning to conceptualize the basic organization of the human self as a distinguishable unit of explanation. This principle is what I will call the individuation *through and from a world*: an individual identity reflects, in its structure and existential needs and concerns, the world *from* which it continuously emerges; but, in order to exist as an individual, it thereby also emancipates itself from the world *through* those very same processes.

This principle demands two things: first, that the processes defining an identity are in principle of the same kind as those of its environment and second, that there is not only interaction with the world but also *emancipation* from it. The two together ground the tension between needing the world (needful) and striving to emancipate from it (freedom).

In line with the hypothesis of the life-mind continuity, I propose to use the principle of individuation *through and from a world* to inspire a new look at the individual self, which can be formalized in terms of the enactive notion of autonomy. The key idea for this to temporarily free Jonas’ notion from the realm of the bodily and organic and to wonder what it would mean for a human *social* individual to be needful and free. The body-social problem for participatory sense-making (and cognitive science in general) arises when, while making the embodied and social turn, one does not fully endorse the principle of *through and from a world*. Freeing, I should thus emphasize, really means to bracket for a moment any role that the body might play in the individuation of human cognitive identity and to instead consider human individuation as a social process rigorously and all the way down (the body does play a non-trivial role but I will get to this in the next section). This means to define the human self *organizationally* as a whole in terms of social interactions and exchanges with the environment. In this context I refer to social interactions as virtual or actual interpersonal engagements of at least two individuals, but also processes of self-relating and being related in social relationships^4^. The types of processes that individuate the self as identity are therefore relational in nature (Tschacher and Rössler, 1996). This realization means that the self is never seen as given or as something that an individual just *has* – it is an achievement, constantly open to change and, at best, something *between* individuals. The self thus never just *is* but rather emerges continuously and jointly relying on behavior and action and on doing and being together with others.

The next important step is to thereby take seriously that, while the principle of individuation *through and from a world* entails the individual’s emergence in dependence on the social world, it also requires its emancipation from it. Without this second aspect, that is without a distinction, the individual would dissolve in social interactions, becoming invisible *as* individual. Again, to introduce this distinction does not require a shift to an ontologically different kind of identity, say the body (or brain). It can be achieved at the same level. It simply means that the social processes involved in individuation matter in different ways: in providing the “material” on which the individual’s identity constructively relies, but also in forming its identity as that particular social individual standing out against the social relations of which it is made. I believe Mead captured the same idea in principle when he said, in *Mind, Self and Society*, that the self is “an eddy in the social current and so still a part of the current” (Mead, 1934, p. 182).

In this way we begin to expand Jonas’ concept of needful freedom, from referring to biological individuation, to an individuation in terms of social interactions. However, to say that the individual emerges through social interactions is not quite enough to capture the idea of freedom and emancipation entailed in the principle of *through and from a world.* Individuation must also involve a particular flexibility and the possibility of ongoing emergence, not just of a one-time instantaneous independence. We have seen this in the case of the organism whose freedom is relative in the sense that it “coincides with their actual collection at the instant, but is not bound to any one collection in the succession of instants” (Jonas, 1966/2001, p. 80). The organism is always dependent on organic matter but what allows it to be an individual organism is that it is not always dependent on the same organic matter.

I propose that just as the organism’s metabolism continuously exerts a choice by taking in only particular processes, while avoiding others, so too the socially organized individual cannot incorporate all social interactions or relations at the same or throughout time, but rather and at different instants in time only particular collections of them. The basic idea is thus to transfer the temporal dimension entailed in Jonas’ perspective on individuation to the level of the human individual and to capture the tension of *through and from a world* by admitting that, while individuation always relies on social interactions and relations, these can vary and matter for the individuation of self to different degrees. In principle the individual does therefore not depend on any single one of them. The construction of human identity occurs not in terms of organismic, but rather *social needful freedom.*

Social needful freedom would do more justice to the role of social interactions and relations than current models of the individual in cognitive science allow: they do not merely matter in that they constitute the individual’s identity as a participant in an interaction or belonging to a group. It is also through social interactions and relations that the individual can free itself and enable itself to move away from some interactions and/or to engage in certain others. Because at different instants in time the individual can engage in certain or disengage from certain other relations, it achieves a relative or functional degree of independency, a mobility that is social. In this way the individual frees and distinguishes itself through time, not merely through being a moving separate body. Nevertheless, as long as it is an individual, it cannot free itself fully from the social interactions and relations, since they are the general “relational material” that it is made of and only against and through which the individual could ever be emancipated^5^.

Let me now indicate how the idea of social needful freedom can be used for elaborating the enactive notion of autonomy as introduced in section “The Enactive Approach to Cognition,” so that it can inspire an approach to self that is integrating without being reductive or essentialist. I would like to emphasize that I aim to initiate the beginning steps toward re-thinking the concept of autonomy to ground novel approaches to the self, not to provide a full-fledged theory of the self.

The model is basic in the sense that it conceptualizes the self at the most encompassing level required for understanding it as an organized unity, while however abstracting over particular phenomena of self, inter- and intra-individual variations, as well as across development, disposition and enactment of self and the particular cultural context, in which the self is embedded in. Indications of how this abstraction can be used to illuminate the different manifestations and dimensions of self will be given later. Right now the goal is to help in avoiding the trap of thinking of the self either as individualistic and embodied, or as social and potentially lost in interaction.

The first step toward a definition of individual autonomy in terms of social cognition is to begin thinking the individual as arising from a sea of social relational, not merely bodily processes. In this way the autonomous network is therefore not only a metabolically “self-generated identity” (Di Paolo et al., 2010) but actually also, and necessarily, an identity that remains open to structural change generated in interaction with others. It is a *self-other-generated* network. This means that the organizational process that constitutes the identity of the individual are defined in terms of interpersonal behavior and action^6^. Let me now determine in a second step just how these processes are minimally organized so that they bring about the individual as self-other generated autonomous network.

**FIGURE 1:**
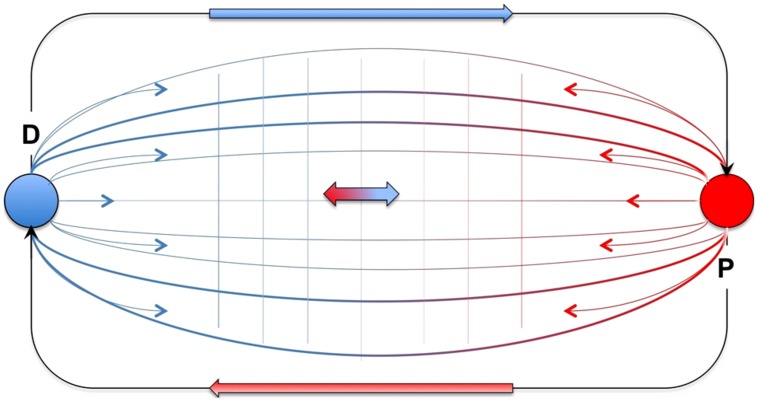
**Socially enacted autonomy.** The graphic illustrates the basic organization of the network of processes that constitute the self as individual socially enacted autonomy. The network processes are social interactions and relations (the blue-red grid) that are spanned between two poles, distinction (blue ball, D) and participation (red ball, P). D and P are interconnected in that they enable each other. Together, the poles determine and qualify the overall tendencies of the network processes (indicated by the blue thin arrows left and the red thin arrows on the right) as having more or less distinction/emancipation and participation/openness. The network processes are in tension (the double arrow in blue and red). When social interactions and relations exhibit higher tendencies toward P, the “pull” from the opposite pole D ensures that the processes do not end up in a extreme degree of P. In this way the network avoids the risk of dissolution. Vice versa, when social interaction and relations have a higher degree of D then the network’s organization tends to balance this with increasing tendencies toward P, thereby avoiding the risk of isolation.

Capturing the idea of social needful freedom in terms of individual autonomy, the autonomous network that constitutes an individual’s self must be organized such that, while principally relying on social relations, it can also resist and therefore free itself from some of these relations. I propose to use the term *distinction* to capture the emancipation as individual *from* certain social relations. Without emancipation there could be no identifiable entity (phenomenologically corresponding to a sense of ipseity, or of alterity in perceiving the other). Being distinct or emancipated however, does not mean that this individual merely stands out, independently, against a vast and unchanging sea of social interactions and relations. In addition to distinction, social needful freedom also entails that the individual continuously becomes individual *through* social interaction and relations. I thus suggest using the term *participation* to denote the other side of social needful freedom: the possibility to organizationally rely at different moments in the succession of time on different instantiations of social interactions and relations (see **Figure 1**)^7^.

Both kinds of network processes, those enabling distinction and those that enable participation, are required together to ensure social needful freedom and to bring about the individual as a network of autonomous self-other organization. Without distinction the individual would risk becoming heteronomously determined and forced to rely on the next best or only a limited set of social interactions. But without participation and its act of openness toward others, the individual eschews structural renewal, thus risking isolation and rigidity. This describes what some enactivists refer to as “precarious conditions” of autonomy (see “The Enactive Approach to Cognition”). In this case, distinction and participation both keep the individual from a particular risk, namely isolation from others or the dissolution in social interactions, and they enable each other in doing so.

I propose to capture these ideas in the following definition for human socially enacted autonomy of the individual:

Individual autonomy is a self-other generated network of precariously organized interpersonal processes whose systemic identity emerges as a result of a continuous engagement in social interactions and relations that can be qualified as moving in two opposed directions, toward emancipation from others (distinction) and toward openness to them (participation).

Because of the tension between a risk to dissolve or to become isolated, the individual, much like the organism, remains permanently concerned with the continuity of its own existence. But while mere living systems strive to survive by avoiding interactions with the environment that threaten their biological survival, the human self *qua* self-other generation has to avoid tendencies in social interactions leading to social death.

**FIGURE 2:**
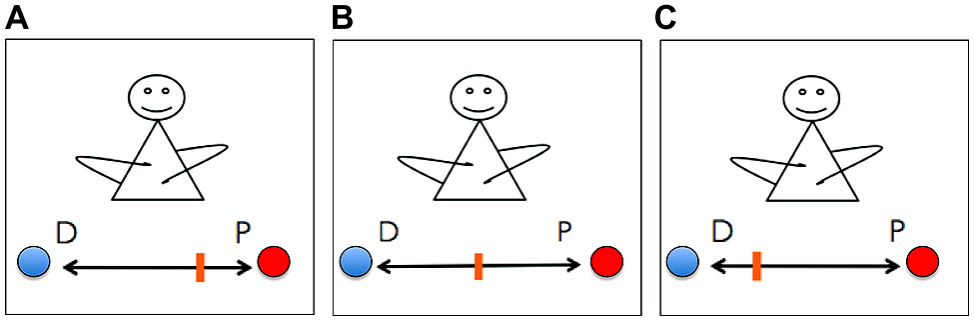
**Adaptive regulation of the twofold basic norm of distinction and participation.** The three graphics illustrate different degrees of distinction (D, blue ball) and participation (P, red ball) in different contexts. Graphic **(A)** illustrates an individual featuring a stronger experience of participation (e.g., when being in love, dancing tango, emerging in the crowd at a concert). Graphic **(B)** illustrates an individual with an equally strong degree of distinction and participation (e.g., in the intimate encounter or during a fight with a close person). The third graphic **(C)** illustrates an individual that experiences a higher degree of distinction (e.g., during a conference talk, in non-transcendental states of meditation).

Just like the organism in its metabolic autonomy, the social human being follows an intrinsic existential norm guiding behavior and evaluations of interactions. The important difference is that the organismic identity as a bodily whole is secured by homeostasis ensuring the body remains stable throughout different interactions with the environment. In the case of the social self, the stability of the unity is not achieved by individual biological or bodily means, but through engaging with others, by learning first how to and then continuously negotiating the balance between the processes of distinction and participation. This balance between distinction and participation is achieved by navigating a range between two extremes, total distinction and total participation and to thereby co-regulate, monitor, identify and seek to avoid tendencies of falling into either of them. This could be the social version of what some enactivists refer to as adaptive regulation (**Figure 2**). The negotiation of distinction and participation can be seen as a co-enacted, quasi-homeostatic principle keeping the self relatively stable and alive as a socially organized and organizing existence.

Mere organismic systems adaptively evaluate their interactions with regards to nutrition needed for the maintenance of metabolism. They seek the right kind and amounts of food, avoiding poisonous food and preferring especially nutritious food. Humans need an additional kind of nutrition. Because human autonomy is co-generated with others, it is necessarily vulnerable to disturbances and conflict. Others can fail or refuse to contribute to a person’s identity affirmation, which could ultimately interfere with the very organizational network that constitutes human autonomy. Particular interactions (or the lack thereof) would lead to problems, either with regards to the individual’s experience *as* somebody individual or with her experiences of being somebody that is *connected* with others. For them to adaptively regulate their own states and interactions with the social environment means to evaluate actions with regard to their contribution to a *socially defined boundary*. To this end, processes enabling or limiting *recognition* of the twofold need for emancipation (distinction) from and openness to others (participation) can be relevant^8^. In line with the present suggestions one could say that social recognition is vital throughout life (Ikäheimo, 2009). Recognition is the nutrient required to co-construct the boundary of the self. If this were not the case, solitary confinement would not be chosen as one of the harshest punishments. As studies with prisoners have shown, social isolation can lead to serious short-term and long-term psychiatric disturbances such as paranoia and hallucinations (Grassian, 1983; Haney, 2003; Guenther, 2013) and as research on social exclusion and ostracism shows human contact is needed to sustain a minimal social identity and prevent social death (Bauman, 1992; Williams, 2007).

According to the present proposal social death has two faces. It could occur when the individual gets stuck in the extremes of either of the two dimensions, distinction or participation. An extreme degree of distinction would mean that the individual has lost its connection to the very structures that it is made from (it risks dying from isolation), while an extreme degree of participation would mean that the individual has lost its individuality (it risks dying from dissolution). There are examples that approximate such extreme degrees in disorders of the self and particularly in symptoms of schizophrenia (Parnass and Sass, 2010), such as social or self-isolation (extreme distinction) or loss of agency (extreme participation).

Recall from section “The Enactive Approach to Cognition” that the enactive approach also provides a route for integrating a third-person, organizational perspective with the subjective dimension and phenomenological perspective of the system itself. Though it is outside the scope of the present argument, a thorough and long-term investigation concerning how the processes of distinction and participation structure subjectivity is as yet required. In the remainder of this section I provide some examples to indicate how humans ensure their survival as social existence through interactions and relations that generate or prevent processes of distinction and participation.

The above definition of socially enacted autonomy proposes that humans co-generate their identity following a twofold norm. This can be used to structure the individual’s perspective on the world in terms of subjective experiences that are evaluated according to whether and how they serve survival, i.e., in this case, the maintenance of the self.

Both distinction and participation are (experienced) types of social interactions and relations, though they say nothing about the amount or actuality of engagement. Distinction roughly means that a person experiences herself as emancipated and distinguished from certain social interactions and relations. It involves a sense of separation and of being someone in her own right. This can apply for a diversity of self-conscious experiences (whether positively, negatively or otherwise evaluated): doing yoga, nervousness in front of an audience, feeling disconnected from your partner, being proud of an achievement, being the stranger at a party, but also the joyful experience of finally being alone after having spent the entire day with other people. Such experiences mirror the basic structure of social autonomy, as striving to maintain a particular degree of emancipation as individual. Participation then generally refers to experiences of feeling both connected and open. It involves a sense of readiness to affect and to be affected by the other. Again, there are manifold examples: the sense of self as curious when falling in love with someone, the pull we feel when finding somebody sexually attractive, a feeling of letting go when dancing tango, being one with the crowd at a concert and so forth. Such experiences refer to the basic structure of social autonomy as striving to remain connected and open to particular types of social interactions and relations (see **Figure 2**).

I have given examples, in which either a sense of distinction or participation is more prominent. However, these two qualities – of experiencing oneself as separate from others and as somebody willing to engage – precede or follow each other, and they can even overlap. There are situations, in which we experience the shift from one quality to another quite clearly. If, e.g., in a difficult discussion our partner finally seems to understand what we want to say, a relief or a sudden relaxation may appear, upon which we begin to feel less separated from the other and begin to experience a readiness to be open again. Yet something about this readiness is already found in feeling separated and misunderstood – one can at the same time feel the need to just overcome the conflict and to be in harmony again. Similarly, at a conference presentation we can experience both a sense of separation from the audience (for instance because of nervousness in the face of criticism) and a sense of eagerness to engage with it (because we would like to discuss our ideas) at the same time. One of the clearest examples of the presence of these two basic kinds of experience is perhaps found in moments of emotional intimacy, or better, in the struggle therein. In an intimate encounter, experiences of wanting to engage and connect to the partner and fear of rejection or of losing oneself are situated very close to each other and individuals can sometimes continuously oscillate between them. In such moments humans can struggle to find the fine attunement between a readiness to let go and be open to other (participation) while, at the same time, an attunement to owning yourself and remaining visible as another individual (distinction). Emotional intimacy is mostly rare, perhaps because it is where the necessarily open and vulnerable self is at its greatest risk.

In contemplation of human existence, it is our task to remind and “elucidate those fundamental aspects that are so familiar to us, so taken for granted, that we often fail to realize their true significance and even deny their existence” (Zahavi, 2008, pp. 127–128). According to the present proposal, what is so familiar to us simply is human life and how it continuously expresses itself to ourselves through sequences of experiences of being more or less separated and of being more or less connected. What we struggle to recognize until we are in a social or personal crisis, in non-transcendental meditation or adopting a researcher’s and philosopher’s stance, is that *both* these experiential dimensions are shades of something that is fundamental to our nature: we need and we want to be individuals in our own right, distinguished, able and free but we thereby also need others and want to be connected, vulnerable, supported and receptive. It is when our standard self-other perception is challenged that we appreciate that these needs are probably never achieved independently from others. Being both emancipated and relational should not be treated independently, both conditions the self at the same time.

This basic model of socially enacted autonomy could constitute an important conceptual move for an enactive approach to the self. It offers an organizational principle for approaching the self as a co-generated and co-maintained whole. On this view, the self is not just a lose collection of aspects but has boundaries that are generated through interacting and being related to others. The self in its most minimal sense, thus escapes the body. It is never fully separable from the social environment, but instead determined precisely in terms of the types of social interactions and relations of which it is, at the same time, a part. Without an ongoing engagement with other people, and without their contribution, there is no generation of self.

Yet, that is not to say that the self is essentially social and “nothing more.” The argument is not in favor of a disembodied conception of the self. To the contrary, as I show in the next section, in this organization of the self as social existence the body plays a more than a trivial role.

## TOWARD RESOLVING THE BODY-SOCIAL PROBLEM

As a consequence of the above proposal, speaking of the embodied self cannot mean that the self *is* the body. Through birth we indeed become a bodily identity, as we “emancipate” ourselves to some extent as physiological entities in a material environment. However, to emancipate as a self, as identity which differs not from organic bodies but *from other human subjects*, a further process of individuation is required (Mahler et al., 2000). This process of individuation, so I suggest in this paper, is achieved through social interactions and relations.

This proposal is fully compatible with the idea of an embodied self where the body, rather than being considered the seat of the self, changes its status and becomes the self’s means and mediator.

The body is then non-trivial for the self as a whole to the extent that it functions as a matrix of co-constructed existence, helping (together with the brain, of course) to organize human social existence and to monitor and regulate the intrinsic goal and minimal purpose of the self: to be some*one*.

It is an open research question how bodily consciousness relates to the human (social) self from an enactive point of view. At this point I can only hint at it. For the enactive approach the creation of a living and cognitive identity brings about a perspective, which is considered as a minimal form of consciousness. This chimes well above mentioned research on the bodily basis of self-consciousness. The idea is to then extend these ideas to the social domain. If, as I suggest, the self is not a bodily but socially co-enacted identity, and if consciousness arises with the creation of identity, then an essential part of (bodily) self-consciousness may emerge through relations with others. Bodily self-consciousness, embodied emotions and existential feelings can then be seen as ways of informing an individual about its state of being in a world of others.

Conjoining the embodied turn with the social in a more than pluralistic sense, the idea of the self as socially enacted continues to do justice to the embodied turn in cognitive science, which recognizes the non-neuronal body, but risks reducing it to a developmental role. It could also pick up where extended functionalist approaches to embodiment remain inflationary (Kyselo and Di Paolo, 2013). Acknowledging that (cognitive) identity is irreducible to the physiology of one’s own body while at the same time considering the body a matrix of an enacted social existence, provides the body with a more clearly defined status. It is not a rock or remote island, but it is also not a random vessel. On the present account, being someone implies being an individual that one can connect to and that remains open to being affected by others. The body plays a major role in making this possible. It is an interface for connection. But the structure of that body interface to the world is not rigid. It is fluctuating, a subject to permanent change – change that mostly happens in reaction to and in dependence on our relations with other beings. In continuation of Bernstein’s theory of motor psychology, according to which bodily movement shapes the brain’s motor system instead of bodily movement being controlled by the brain (Thelen, 2004), within the logic of the argument at hand, there might be a further reversal regarding the relation between body and sociality. The body is not merely a means but also an imprint of social engagement. As a consequence, bodily consciousness alone would be insufficient to ground even the most minimal sense of the human self. Instead, it might be seen as a kind a sensor for monitoring social engagements and relations with the goal of social homeostasis. This sensor does not merely reside within the realms of the individual’s body and actions, it is also co-constituted in and through the space created between individuals.

Of course, there is something quite crucial to insisting that a person feels their very self changes when they change bodily aspects of their existence, be it when they become sick, suffer an accident leading to disability or even when they only change slightly, say with getting a new hair cut or dress. But we can admit this without also arguing that body and self are ontologically the same. The point I want to make is that many bodily changes matters for someone because of what they mean with respect to this person’s relation to the social world and how she fares in its relation to others. Bodily experiences acquire a social meaning and I propose that this meaning is generally evaluated according to the twofold norm of distinction and participation. The new hair is not merely a change to some biomaterial that grows out of my head. It is a change to the way I look, and thus relate to myself and to others, and of course to the way, in which others relate to me. My partner might notice the difference in style and compliment that I look fresher, more beautiful etc. But if after my haircut I went to work for *medicins sans frontiers*, the change of style would probably not matter much. The point is what I feel about my haircut depends on how I saw and now see myself and on how others have seen and now see me. It requires an implicit act of relationality to make this bodily change significant for my self.

Let me now come back to the two empirical examples, introduced in section “The Body-Social Problem in Cognitive Science” where this point becomes more pressing: the possibility of positive quality of life in LIS and social pain. Recall first the case of LIS patients, who despite being globally paralyzed, report a positive quality of life. One way of making sense of this is by adopting what I would call a cognitive adaptation strategy. In a recent study, Nizzi et al. (2012) conducted interviews with LIS patients to assess how the paralysis affected their sense of personal identity. They found that patients can adjust very well to the objective change in physiology and actually “feel the same as before the accident.” According to the authors, this is because the patients maintained a positive subjective “bodily representation” (p. 435). If positive quality of life has to do with a positive self representation then this adjustment strategy can explain why patients feel well despite the paralysis. However, Nizzi et al.’s (2012) interpretation seems to presuppose a disembodied view of the self. Whether or not the body is subject to severe objective change plays no role for the patient’s self as long as she consciously decides that it does not. One of the problems for an explanation of well-being in LIS is that it risks trivializing the role of the non-neuronal body for the self – all the necessary work could be done by a bodily representation, presumably located in the brain. For an (essentially) embodied approach to the self this interpretation must seem counter-intuitive. The embodied self implies that there is a relation between objective physiological change and subjective experiences of self and well-being. On adopting this view, one would probably have to assume that LIS, being a global bodily paralysis, is in a sense also a disorder of the self and of (bodily) self consciousness. If the self is equated with the body and the bodily self considered as grounding first-person subjectivity, then the patients’ well-being should be affected. And yet, as the results of Nizzi et al.’s (2012) interviews and other qualitative studies on LIS patients seem to suggest, this is not the case. The embodied approach to the self (as a whole) would thus actually make a counterfactual prediction.

The proposed model of the self as socially organized autonomy could provide an alternative to the cognitive adaptation story. On the enactive interpretation, the self remains non-trivially embodied in the sense that it is mediated by the body; the body is part of the interface organizing the individual’s social existence. According to this perspective, the patient can adapt to the new situation precisely because she is not the physiological body, but a genuinely social self. The physiological change matters because it changes the ways, in which the patient is able to relate to others and, in which others relate to her. To the extent that these relations are still given, even the most minimal form of communication – as can be seen in the usage of brain computer interfaces – can suffice to enact the processes necessary for the individuation of self (distinction and participation) and thus for integrating bodily changes into a positive sense of self. This interpretation is also empirically supported by studies of less severe forms of disability. Babies with Moebius syndrome, for example, lack facial expressions and are unable to show their care-givers “that someone is home” (Cole, 2009, p. 351). This can affect how care-givers react to their children. They might respond to them with “reduced signals” which can in turn cause “emotional impoverishment” (Cole, 2009, p. 354). For patients with spinal cord injury “disablement [ha]s nothing to do with the body. It is a consequence of social oppression” (Cole, 2009, p. 348). Paralysis is “not simply a physical affair ... but an ontology, a condition of our being in the world” (Murphy, 1990, p. 90). Despite global restrictions, the LIS patient is still “yearning for intersubjectivity” (Dudzinski, 2001, p. 43). Statements such as these suggest that it is through being related to others that bodily changes can affect and be integrated in our self. The fact that the “quality of life often equates with social rather than physical interaction” (Gosseries et al., 2009, p. 199) makes sense when the boundaries of the self are not determined by bodily processes alone, but rather in terms of relational and co-enacted processes. LIS can be considered a disorder of the self to the extent that the body is restricted as the individual means of social relationality, not as the seat or constitutive basis of the self. More accurately, like other cases of disability, LIS should be seen as a “disease of social relations” (Murphy, 1990, p. 4). This also means, for better or worse, whether she is able to integrate severe bodily changes and lead a happy life, does not entirely depend on the patient herself, but also on the support and recognition of others.

An interpretation of well-being in LIS makes sense from a disembodied view, but the idea of the self as mediated by the body offers a non-reductive explanation, doing justice to both, the embodied and the social turn in cognitive science.

The present proposal also makes sense in light of the fact that social rejection hurts (see “The Body-Social Problem in Cognitive Science”). One might be tempted to read this fact prima facie as evidence for the primacy of the organic body in individuating the self as a whole and so as supporting the idea of the (essentially) embodied self. This is indeed what Eisenberger seems to have in mind when arguing that the pain is evolutionary beneficial since it helps to ensure survival. On such a reading, the social matters, contextually in allowing an individual to survive as a biological identity (a minimal bodily self, if you will). The social rejection of being excluded from participating in a game hurts because it indicates a risk, namely that others will not be there to help protect the biological self^9^ .

The alternative would be to consider the evidence that the major source of concern for human existence does not stem from nuisances within the organic body itself, but rather from the fact that human existence is organized socially. Thus, instead of reducing sociality to the role of the means to a biological end, why not take the evidence as direct support for the fact that humans are concerned about their existence as social beings? I would agree with Eisenberger that the pain of social rejection is beneficial for survival. But in light of the present consideration, this survival is not merely biological. Rather, the empirical example can be seen as support for the hypothesized relation between socially enacted autonomy and the fundamental role of social recognition as enabling the processes of distinction and participation. Social rejection constitutes a potential violation of recognizing me as someone others can connect to or who can connect to others, but it also risks reducing my ability to be seen as a distinct individual. On assuming that the body mediates a socially enacted self, pain of social rejection could be one of the body’s clever ways of cautioning the self against the lack of recognition and its ultimate consequence, *social* death. I would thus reverse the standard argument: the social does not help the bodily self as a whole, instead the body is helping the self to survive as a social whole.

To conclude these considerations on the quality of life and pain of social rejection, there is no logical reason that forces us to prefer one of the three possibilities of interrelating body, self and sociality (disembodied, essentially embodied or bodily mediated). The first example supports both a disembodied and a socially enacted view of the self, while the second example seems to be plausible on both an essentially embodied and on a socially enacted and bodily mediated account of the self. I am thus not arguing that my approach is the only game in town. What I would like to suggest however is that it might be preferable for the purpose of cross-disciplinary dialog, since it rises to the challenge of the body-social problem without avoiding either, the embodied or social turn in cognitive science. At the same time it might have advantages over a pattern approach to the self, since it does not merely account for diversity but also provides an account of the self as a coherent unity and determines how other dimensions such as sociality and (neural and more than neural) embodiment might integrate as aspects of this unity.

## CONCLUSION

In this paper, I have introduced the body-social problem, the question for cognitive science of how bodily and social aspects go together in an account of the human self as a whole. I have discussed the problem in more detail with regards to research on social cognition in enactivism, where it translates to the question of how bodily individual autonomy and higher, socially enacted forms of autonomy, are interrelated.

I proposed the principle of individuation through and from a world to extend Jonas’ notion of needful freedom and to ground an integrative perspective on the embodied and social self. According to this principle, humans emancipate themselves not merely through organic, but also interpersonal, interactions. Their identity emerges out of a tension concerning social freedom: humans strive to distinguish themselves from others as individuals, yet at the same time they also strive for connection with, and being affected by, others.

I elaborated on the enactive approach to individual autonomy and indicated how this discussion can inform an approach to human identity as co-generated and organized in terms of an adaptive regulation of social distinction and participation processes. I have argued that the enactive approach to the self can be a way for cognitive science to avoid the dilemma of the body-social problem. One does not have to choose between positing an isolated bodily individual or an individual as mere participant. The positive contributions entailed in both horns of the body-social dilemma are brought together in an integrative way. In this view, humans are participating and therefore able to emancipate themselves, and because they emancipate themselves they are able to participate. The self is constitutively social, not merely developmentally, but throughout its life. The body’s role is to mediate that social existence and is the major key to ensuring the twofold goal of maintaining both distinction and participation, leaving the possibility open for non-physiological forms of self-co-maintenance, using tools and language-based technology.

The paper provides an alternative to a pattern approach to the self. It acknowledges diversity but as shown in the context of empirical examples, such as the positive quality of life in patients with global paralysis and the pain of social rejection, it also offers some ideas for how they integrate.

These considerations are not meant as a final word on the question of how self, body and sociality interrelate. The paper provides some novel and basic conceptual suggestions for cognitive science to integrate embodiment and sociality in a way that neither underestimates the role of interpersonal relations, nor runs the risk of losing the individual through an overemphasis on group and interaction dynamics. I propose them as stepping-stones toward a biologically based, yet social and experientially plausible approach to human individuation. Further investigations, to this end, are required, including philosophical inquiries on self and intersubjectivity at the intersection of philosophy of mind and phenomenology as well as philosophical anthropology. Further required are explorations of existing linkages to intersubjective approaches to self and subjectivity in other fields of cognitive science, especially developmental psychology, psychiatry, and social neuroscience.

## Conflict of Interest Statement

The author declares that the research was conducted in the absence of any commercial or financial relationships that could be construed as a potential conflict of interest.

## Footnotes

1 I borrow this image from Varela et al. (1993) who used it not in a social sense but in support of the idea that the organism and its environment co-determine each other. The authors caricatured the cognitivist view as implying that the environment is a “landing pad for organisms that somehow drop or parachute into the world” (p. 198).

2 The body-body problem is the question how a living body can bring about embodied experience (Hanna and Thompson, 2003; Thompson, 2007, pp. 235–237).

3 I rely on a recent distinction made by De Jaegher et al. (2010) between *contextual*, *enabling,* and *constitutive* roles of the social for cognition.

4 My requirement for an interaction to count as social is therefore lower than typically assumed in enactive social cognition. A social interaction need not involve equal subjects. A relation between an infant and its care-giver, a prisoner and guard or between an ego-centric and empathic person is social even when the recognition of subjects as free and autonomous individuals might have different degrees.

5 The idea of social needful freedom might be in tension with some phenomenological accounts of intersubjectivity (e.g., Henry, 1988 or Husserl, 1992/1930). The sense of self in our self-other relation involves a first-person givenness and therewith a sense of separation from others and, at the same time, there is something about the experience of the other that escapes my own experience. This seems to contradict my idea that the individual can never free itself from the social as material of its own self-constitution. I am afraid I cannot do justice to the rich body of phenomenological inquiries into self and understanding others in this paper. But generally speaking I am convinced that the idea of social freedom is in principle compatible with phenomenological accounts of intersubjectivity. What it challenges are some deep intuitions about the structure of first-person perspective and to which extent experiences of ipseity and alterity are conclusive to it. The suggestion that, contra Henry’s absolute immanence, it might be relationally co-enacted, is not to replace, but rather to complement phenomenological inquiries. My hope is that the proposed *organizational* perspective herein helps to specify just the how first-person perspective is constituted. This could be seen as part of an ongoing dialog between cognitive science and phenomenology of intersubjectivity.

6 Such overlap of behavioral and identity constructing processes can already be seen in non-human animals. Fisher spiders for example store air bubbles trapped on the surface of their body, thus mechanically stabilizing the bubbles (so called “plastrons”) to prevent them from collapsing under water. They then use the oxygen contained in the bubbles to survive for longer periods than they otherwise could, under the water surface (Flynn and Bush, 2008). Thus the survival of the insect under water relies not only on constructive processes (of its metabolism) but also on a particular behavioral and interactive strategy (collecting bubbles, storing them etc.).

7 Note that social relationality need not translate to actual engagement, or actual interaction, with others. The self in the mode of *distinction* is not suddenly socially unrelated, rather it is socially related in two ways: first, in order to be distinguishable as individual it relies on particular social relational processes that favor its distinctiveness, and second, it is related in that the social relational processes that do not favor or do not matter for its distinctiveness necessarily act as the system’s environment only against which is visible as unity. Similarly, being in *participation* does not mean to cease to exist as separate individual; were this the case, no *one* would be distinguishable as participant. For this reason participation is not equal to participatory sense-making. The latter adopts a more narrow perspective on the individual, which is determined heteronomously, in terms of its contribution to a group identity.

8 An important question for further elaboration is how processes of distinction and participation could be mediated in linguistic terms. To this end, it might be fruitful to relate the present argument to Maturana’s work on languaging and the creation of consensual domains in which individuals co-structure their social, not merely organismic, identities (Maturana, 1978). A further crucial linkage exists to developmental psychology. Research showing the vital role of intersubjective engagement in early infant development (e.g., Trevarthen, 1993; Braten, 2004; Stern, 2009) could be relevant for specifying how processes of distinction and participation organize the initial development of socially enacted autonomy. The educational psychology of Bruner, who was also the first to use the term “enactive,” could inspire further elaborations of how children continuously expand their self-reflexive capacities and understanding others through active, intersubjectively structured learning (Bruner, 1996).

9 One might wonder whether and to what extent this can be extrapolated to human identity in general. It could invite an odd argument according to which humans must suffer physiological pain for every social activity in which they are not included. This is obviously not the case. Whether a certain interaction counts as a case of social rejection might be better determined by evaluating whether it means something to the person, and this depends on how she is related or wants to be related to the people involved in the interaction. If a person does not care to be included in the activity, then she would not feel rejected and therefore also not experience physiological pain. Even if the person desires to be included, if she reassures herself that that the exclusion is temporary, she avoid interpreting the situation as a rejection and thus remain pain-free.
